# Association of melatonin receptor 1 A with litter size in sheep: A review

**DOI:** 10.12688/f1000research.134890.2

**Published:** 2024-01-22

**Authors:** Mutasem Abuzahra, Dwi Wijayanti, Mustofa Helmi Effendi, Imam Mustofa, Mirni Lamid

**Affiliations:** 1 Veterinary Medicine, Universitas Airlangga, Surabaya, Indonesia; 2 Department of Animal Science, Perjuangan University of Tasikmalaya, Tasikmalaya, West Java, 46115, Indonesia; 3 College of Animal Science and Technology, Northwest A&F University, Yangling, Shaanxi, 712100, China; 4 Department of Veterinary Public Health, Faculty of Veterinary Medicine, Universitas Airlangga, Surabaya, Indonesia; 5 Department of Veterinary Reproduction, Faculty of Veterinary Medicine, Universitas Airlangga, Surabaya, Indonesia; 6 Department of Animal Husbandry, Universitas Airlangga, Surabaya, East Java, Indonesia

**Keywords:** sheep; MTNR1A; litter size; polymorphism

## Abstract

Sheep are a valuable livestock species worldwide, providing meat, milk, and various dairy products. This article aims to review the latest literature on the melatonin receptor 1A (MTNR1A) gene as a potential candidate gene associated with reproductive traits, particularly the litter size trait in sheep, by searching various databases for available literature. Studies have shown that different parts of the MTNR1A gene play various roles in sheep. By identifying marker genes associated with reproductive traits in MTNR1A polymorphisms linked to the litter size trait, breeders can achieve a faster selection response in sheep breeding by recognizing the genomic region where these genes are located and understanding their physiological functions. Therefore, highlighting the literature on these functions and their association with reproductive traits may contribute to improving the genetic makeup during sheep breeding.

## Introduction

As a result of the increasing demand for livestock production, there is a growing need for animals that are able to reproduce larger litter size. ^1^^,^^2^ The ovulation rate, hormones, and growth factors all play a role in determining the litter size, which is one of the most important characteristics of fertility in animals. ^3^^,^^4^

It has been noted that in sheep breeds where estrous activity peaks during short days, variations in day length may function as a main factor determining seasonal variations in estrous activity. ^5^^–^^7^ In the pineal gland, the indole hormone melatonin (MT) is produced, ^8^ which is linked to changes in light signals of the external natural environment. ^9^^–^^13^

The binding melatonin receptor, also known as MTNR, has a variety of biological purposes, some of the most important of which include the regulation of animal sexual behavior, reproduction, and circadian rhythm ^14^ a factor that has a positive influence on reproduction even when the night is longer. ^9^ In most livestock species, mutations in the *MTNR1A* gene are linked to ovarian function and litter size. ^12^^,^^15^ Despite the fact that several scholars have investigated the aspects of growth that influence the reproductive characteristics of goats. ^16^^,^^17^

Melatonin has an effect on reproduction, which is mediated through receptors that are found in the hypophyseal pars tuberalis. ^18^ Melatonin has an effect on the pars tuberalis, which in turn has an effect on the seasonal reproductive activity of females in a number of different mammalian species. ^19^ This includes the nuclei that are responsible for reproduction. ^20^ Preceding studies have revealed that the polymorphisms in the *MTNR1A* gene sequence have an effect on the ability of Rasa Aragonesa rams to reproduce both when they are young and when they are adults. ^6^ In particular, the T/T or G/G genotype was shown to be connected with earlier ram-lamb mating activity, and adult rams that carried the T/T or G/G genotype exhibited the highest reproductive behavior in the spring. ^20^

Due to the fact that sheep have a clearly established circadian rhythm, ^10^ it is vitally necessary to investigate the mechanisms behind seasonal reproduction in this species. Melatonin production can be stimulated by exposing the eye to less light for longer periods of time. The pituitary gland’s production of follicle-stimulating hormone and luteinizing hormone is influenced by the amount of melatonin that is present in the bloodstream. ^10^ The purpose of this article is to review the most recent literature on this topic may help to shed light on the mechanisms behind seasonal reproduction in sheep.

## *MTNR1A* location

The gene known as *MTNR1A* can be found in sheep on chromosome 26. It involves two exons that are split up by an intron that is nearly 22 kilobases (kb) in length and stretches from the position 17,354,820 to the position 17,377,973 in the Oar rambouillet v1.0 genome assembly. This region can be found between the two numbers 17,354,820 and 17,377,973. The length of exon I of the *MTNR1A* gene is approximately 260 base pairs, whereas the length of exon II is around 970 base pairs. ^7^^,^^21^ Ewes’ seasonal reproductive activity is linked to the presence of many polymorphic sites at the *MTNR1A* locus, which can be found to be variable. ^5^^,^^22^ Despite the fact that the ramifications of the *MTNR1A* gene on reproductive seasonality have only been investigated to a limited extent up until this point, those studies have shown that these effects are tied to three specific mutations discovered in exon II at locations 606, 612, and 706, ^7^^,^^21^ only sheep of the Sarda and Aragonese breeds have had the remaining portions of this gene examined. ^12^^,^^13^^,^^23^^–^^25^

## Mechanisms Involved in the Production and Secretion Control of Melatonin

The pineal gland predominantly secretes melatonin during nocturnal hours, thereby designating this hormone as an inherent informant of the photoperiodic trend. ^26^ The circadian influence of melatonin is orchestrated through melatonin receptors located in the hypothalamic suprachiasmatic nucleus, the central hub of the circadian clock. Concurrently, the reproductive impacts of melatonin transpire within the premammillary hypothalamus. Melatonin exerts both its circadian and reproductive effects by engaging high-affinity G-protein coupled receptors. ^26^^,^^27^

The endocrine signal and circadian rhythm of melatonin secretion play a pivotal role in mediating the influence of photoperiod on the hypothalamus-pituitary axis, significantly contributing to the regulation of seasonal reproduction. ^7^^,^^27^ The reproductive impacts of melatonin are orchestrated through ligand-specific guanine nucleotide-binding protein (G-protein) coupled receptors situated in the hypophyseal pars tuberalis. ^27^ Retinal reception of light signals initiates the synthesis of melatonin by the pineal gland. In sheep, elevated melatonin levels coincide with a rapid increase in darkness (short photoperiod), thereby stimulating the pulsatile secretion of gonadotropin-releasing hormone (GnRH) and subsequently resulting in the secretion of luteinizing hormone (LH). ^28^

Melatonin, typically synthesized in the brain but also capable of secretion by granulosa cells, serves as a recognized modulator influencing follicle development, oocyte maturation, and embryo development. ^29^^,^^30^ In sheep, melatonin-treated ewes exhibit enhanced follicle development in culture compared to their untreated counterparts. Additionally, melatonin serves to modulate gene expression associated with steroidogenesis, differentiation, and luteinization of granulosa cells. ^31^ This hormone safeguards the integrity of oocytes and granulosa cells through its specific receptors, MTRN1A and MTRN1B2, by actively scavenging reactive oxygen species (ROS) and regulating apoptosis-related genes, thus preventing apoptosi. ^32^

## *MTNR1A* role by season

The duration of nocturnal melatonin secretion is affected by photoperiod variations between short and long days, which controls reproductive activity through the hypothalamic-pituitary-gonadal axis in seasonal sheep breeds located in areas with a temperate climate. ^26^ These sheep breeds are bred in climates that are considered to be moderate. ^33^ In the study of Martínez-Royo ^25^ observed that reproductive functions were present in Merino d’Arles only in the month of April, as evidenced by varying progesterone concentrations. It was also shown that the single nucleotide polymorphism (SNP) known as rs430181568, which was formerly known as 612/MnlI, is connected with the reproductive patterns that are associated with the different seasons. When analyzing the patterns of ram-ewe mating in the Rasa Aragonesa breed, ewes with the A/A genotype at the rs406779174 gene showed higher fertility between January and August than those with G/A or G/G genotypes. Ewes mating to rams or other ewes had the same results. ^13^ This was the case regardless of whether the ewes were mated to rams or other ewes.

Both of these (606 and 612) loci have been linked in Indian breeds to ewes having lambs during times of the year when the ewes of these breeds would not normally be in oestrus. ^19^ These two SNPs were found to be associated with the resumption of reproductive activities in the fall, following a period of seasonal anoestrus, in the animals studied, Slovenia is home to a variety of sheep breeds used for both dairy and meat production. ^15^

Melatonin production requires the participation of two distinct receptors; however, simply the *melatonin receptor subtype 1A* ( *MTNR1A*) is believed to be a potential candidate gene that mediates the photoperiodic reproductive seasonality in sheep. ^19^ Certain genotypes have been linked to reproductive activity that occurs outside of the normal breeding season. ^13^ Despite the fact that sheep milk is a highly significant produce for Greece and the raw material intended for a great deal of products with a protected designation of origin. ^9^ Polymorphisms found within the *MTNR1A* gene of the Sarda sheep breed, which has a period of anoestrus in the late winter/early spring, led to improvements in the return of reproductive function. ^7^ However, in the Aragonesa breed, the polymorphism at position 612 was the sole one linked to a larger percentage of oestrous cyclic ewes between January and August. ^13^

The change in annual photoperiod, which governs the production of melatonin and is the primary environmental element that determines reproductive timing. ^15^ Melatonin secretion is lower throughout the long photoperiodic phases of the year, whereas it is higher during the shorter photoperiodic periods. As a result, various biological rhythms exist, such as seasonal reproduction, which is regulated by photoperiodic patterns. ^15^

In small ruminants, the considerably higher nighttime MT concentrations that generally occur throughout the fall have a beneficial influence on reproductive activity, ^15^ Allelic variations at locations g.15099491C > T and g.15099485 A > G in the *MTNR1A* gene exon II have been connected with the seasonal reproductive features in a variety of sheep breeds. These sites are located in the *MTNR1A* gene, ^8^^,^^9^ only the g.15099491C > T polymorphism was linked to a greater proportion of oestrous cyclic ewes in the Aragonesa breed between January and August. ^13^ These findings suggest that there is a connection the *MTNR1A* gene and reproduction season. Breed, management (including the presence or absence of males), and environmental conditions can all affect the timing of a gene polymorphism and the subsequent reproductive season. Thus, the length of time researchers observed subjects before drawing conclusions about the relationship between polymorphisms of this gene and the resumption of reproductive behavior is likely to be variable between studies. ^22^ The findings of all of this research suggested that there was a relationship between the *MTNR1A* gene and the modulation of seasonal impacts on the reproduction of ewes in the breeds that were investigated. ^13^

## *MTNR1A* in female reproduction

After ovulation, progesterone is crucial for both the maintenance of pregnancy and the maintenance of a normal estrous cycle. As a further point, the granulosa cells in bigger ovarian follicles secrete the most progesterone. ^34^ Because melatonin causes an increase in the diameter of the corpus luteum, it also causes an increase in the concentration of plasma progesterone. ^35^ Melatonin might well be able to exercise direct control over the ovary due to the existence of melatonin receptors in ovarian follicles. ^36^ Melatonin has been shown to stimulate progesterone secretion in granulosa cells, and it appears that *MTNR1A* plays a role in this process. However, blocking this receptor significantly reduces progesterone synthesis, melatonin’s effect on progesterone production may be partly due to *MTNR1A*, Consequently, melatonin’s ability to affect reproductive behavior in sheep stems from its ability to inhibit apoptotic genes while simultaneously stimulating antioxidant genes and progesterone synthesis via the MT1 receptor ( *MTNR1A*). ^37^

Due to the fact that melatonin is a hormone that is secreted by the body, its influence on the development of sheep oocytes in vitro is an excellent subject for research into the underlying molecular process. This was demonstrated by an experiment conducted in vitro, which also discovered that the addition of MT to the maturation process in vitro boosted the percentage of cultivated sheep oocytes that developed into blastocysts. The most valuable application of sheep MT is in scientific research. ^38^

## MTNR1A gene and the reproductive performance of rams

It is unclear whether the specific gene variations responsible for the seasonal pattern in male sexual behavior have any impact on ram reproduction. Melatonin, produced in the testes, interacts with melatonin receptors in various testicular locations. ^6^ The reproductive behavior of female ewes is influenced by breed, season, and the presence of male rams. However, the application of uniting males with ewes in breeding populations is challenging due to the variable reproductive responses of ewes ^33^ Melatonin receptors MT1 ( *MTNR1A*) and MT2 ( *MTNR1B*) in the testes, accessory glands, and sperm suggest a direct effect of melatonin on the ram’s reproductive tract. ^32^

Studies indicate that melatonin can be produced in the male reproductive tissues, particularly the testes, explaining the high levels found in ram seminal plasma, especially during the daytime. ^38^ Melatonin concentrations in seminal plasma and follicular fluid can exceed those in blood serum, suggesting a direct influence on sperm function. ^39^ Melatonin affects sperm capacitation and in vitro fertilization outcomes in rams. The effects depend on the dosage, with low doses promoting capacitation and high doses inhibiting it. ^39^ Melatonin acts by reducing cyclic adenosine monophosphate (cAMP) levels, suppressing protein kinase A (PKA) activity, a pathway involved in sperm capacitation. ^40^ Different subpopulations of ejaculated ram spermatozoa exhibit variations in melatonin receptor distribution, suggesting differences in their physiological states. Melatonin inhibits apoptosis and modifies sperm capacitation through the MT2 receptor. ^39^ Melatonin produced in the testes protects developing spermatozoa from oxidative damage, additionally, seasonal changes in ram seminal plasma composition and the impact of melatonin treatment have been observed. ^35^

Melatonin implantation in mature rams stimulates testicular growth, increases testosterone levels, improves semen quality, and enhances overall reproductive performance. ^41^ Melatonin directly affects sperm motility and other characteristics during non-breeding seasons. ^41^ Administering melatonin during the non-breeding season can improve testicular function beyond signaling the onset of the breeding season. Effective doses for enhancing testicular size in mature rams range from 36 to 54 mg. ^41^ Furthermore, melatonin protects testicular tissue and improves semen quality in heat-stressed male goats by reducing oxidative stress. ^42^

## *MTNR1A* function

In animals, two high-affinity melatonin receptors known as MT1 and MT2 have been discovered; nevertheless, only MT1 is important in the regulation of reproductive behaviors. Melatonin exerts its effects by binding to certain receptors that are situated in a number of organs, the nuclei of the central nervous system are responsible for regulating reproductive activity. Melatonin’s actions are exerted. ^15^

Although the Pars Tuberalis (PT) contains a significant number of receptors, this formation is only slightly implicated in the regulation of reproduction. ^5^ However, the PT unquestionably plays important roles in the photoperiodic regulation of prolactin secretion. ^16^ However, only a tiny number of these receptors are found in the premamillary hypothalamus, which is the area of the brain responsible for reproduction when melatonin is present ^15^ is only slightly implicated in the reg. ^15^ The MT1 receptor gene ( *MTNR1A*) has been found to exist in a variety of organisms. It belongs to the class of proteins called G protein-coupled receptors. There are a variety of polymorphisms, in exon II of the *MTNR1A* gene, that are known to influence the reproductive capabilities of different species according to the changing seasons. ^43^

*MTNR1A* and *MTNR1B* are the two subtypes that can be distinguished when referring to melatonin receptors. The suprachiasmatic nucleus and pituitary nodules of the hypothalamus are the primary locations where *MTNR1A* is found in animals. ^8^ This location is significant since it is associated with the control of animal reproduction. ^8^ The activity of *MTNR1B* is not particularly high, however *MTNR1B*-mediated MT may have an effect on insulin secretion. ^44^ Therefore, MT mostly joins with *MTNR1A*, performing a particular function in the biological world. ^23^

Melatonin activates hypothalamic receptors. The suprachiasmatic nucleus houses the circadian clock, and thepremamillary hypothalamus controls the hormone’s reproductive actions. ^13^ The premamillary hypothalamus has less melatonin MT1 receptors than the pars tuberalis. ^13^ Sperm from non-seasonal animals have melatonin receptors. Their roles in spermatozoa may not be tied to seasonal fluctuations. ^39^ Protection of human sperm from oxidative damage and apoptosis has also been linked to the melatonin receptor MT1, ^45^ MT2, on the other hand, has been linked to the regulation of the capacity of male sperm to reproduce in vitro. ^46^ The plasma of rams from three different sheep breeds was shown to contain melatonin when the rams were exposed to an equatorial photoperiod that changes between wet and dry seasons, ^47^ while the source of this variation persists unidentified. ^32^

## Genetic association in the *MTNR1A* gene

Multiple variants of the MTNR1A gene have been discovered in sheep breeds worldwide, some of which have little impact on reproductive ability, while others can improve ovulation rates and litter sizes, or play an important role in male reproduction.

He *et al.* ^3^ conducted an association analysis that revealed a notable influence of the mutation at g.15118756C > T on litter size (first, second, and third parity) in Small Tail Han sheep (p < 0.05). This observation suggests a fundamental contribution of polymorphisms in the ovine MTNR1A gene to the variability observed in sheep litter size. Three single nucleotide polymorphisms (G735A, G753A, and C845A) were detected in both Chinese Merino and Prolific Suffolk sheep, and an association study revealed a significant association (p < 0.05) between these three SNPs and litter size. ^48^

Kianpoor *et al.* ^49^ demonstrated a statistically significant relationship between the morphological characteristics and the genotypes of the MTNR1A gene. However, according to the results of Fathy *et al.*, ^50^ the MTNR1A gene polymorphisms were not associated with most characteristics related to sperm quality and testicular size. Instead, the Ossimi and Barki breeds had the highest frequency of allele C, while the Rahmani breed had more allele T, with both Rahmani and Ossimi ewes with the MTNR1A SNP showing the shortest age at first lambing. Similarly, Mura *et al.* ^51^ showed that rs430181568 and rs407388227 were linked to the fecundity of adult Sarda ewes. Moreover, the MTNR1A gene affects ram fertility in both juvenile and adult stages, as seen in the results of Vibha *et al.* ^52^ and Mura *et al.* ^23^ However, some studies, such as that of Z. Davari Varanlou *et al.*, ^53^ did not find any association between this gene and reproductive or growth characteristics.

The study discerns noteworthy correlations between specific SNPs and haplotypes, establishing significant associations with enhanced reproductive recovery and a reduction in days to lambing in Awassi ewes. A comprehensive analysis identified a total of 31 SNPs, inclusive of five missense mutations. Haplotypes H2, H3, and H4, alongside SNPs rs430181568 and rs40738822721 (hereafter referred to as SNP20 and SNP21), demonstrate a robust statistical correlation with decreased days to lambing (p < 0.05). Importantly, SNP20 and SNP21 exhibit complete linkage, functionally consolidating into a singular marker. Ewes harboring the C allele at both polymorphic loci exhibit a significantly expedited reproductive recovery (p < 0.05). These findings bear paramount significance for refining strategies in reproductive management and attaining heightened lambing efficiency in Awassi ewes. ^22^

The study definitively establishes the impact of specific nucleotide sequences within the MTNR1A gene on reproductive functions in Mediterranean sheep. Distinct genotypes at specific gene positions correlate with increased fertility and reduced duration from ram placement to lambing across diverse sheep breeds. Particularly, SNPs at positions g.17355452 and g.17355458 significantly influence reproductive performance. In Bovska and Sarda, C/C and C/T genotypes at g.17355452, and in Istrian Pramenka, A/A genotype at g.17355458, are linked to heightened fertility and shorter duration to lambing. These findings underscore the influential role of MTNR1A gene sequences in shaping reproductive outcomes in Mediterranean sheep. ^13^

Further research suggests that both MTNR1A SNPs, g.15118683C > T and g.15118951G > A, may influence the timing of oestrus and pregnancy in certain sheep breeds, with Small Tail Han sheep carrying a homozygous mutation (TT) at the g.15118756C > T locus exhibiting higher litter size. ^7^ However, no correlation between genotype and litter size was. ^1^^,^^2^ The ovulation rate, hormones, and growth factors all play a role in determining the litter size, which is one of the most important characteristics of fertility in animals. ^3^^,^^4^

Mura *et al.* ^23^ reported the use of RsaI and MnlI restriction enzyme digestion to identify T606C and A612G polymorphisms in the amplicons. Ewes with the G/G, G/A, C/C, and C/T genotypes exhibited increased fecundity (p < 0.05) and shorter intervals between ram introduction and parturition (p < 0.05) compared to those with the A/A and T/T genotypes. The study revealed that mutations in the MTNR1A gene affected the timing of the Sarda sheep breed’s return to reproduction in the spring. Furthermore, variations in the MTNR1A gene sequence impacted ram fertility during both juvenile and adult stages of life. T/T and G/G ram-lambs born in the fall had superior reproductive abilities, while T/T and G/G adult rams exhibited the most aggressive mating behavior. ^6^ The SNP rs403212791, which alters the amino acid content from Arginine to Cysteine (R336C), may result in reproductive seasonality trait changes. The rs403212791 T allele in the MTNR1A gene was positively associated with the oestrus cycling months OCM values, which indicate behavioral indicators of oestrus in the Rasa Aragonesa breed, and negatively correlated with TDA values, which reflect seasonality attributes for ovarian function based on blood progesterone levels. ^21^

Thus, it is crucial to further investigate the true involvement of MTNR1A through additional research with larger sample sizes and a wider variety of sheep breeds from around the world, as well as the mechanism of its SNPs in reproductive traits. MTNR1A gene has a significant effect on the reproductive traits of various sheep breeds. Different alleles and genotypes of this gene have been linked to variations in fecundity, timing of sexual maturation, litter size, and body weight. However, there is still a need for further research to confirm these findings and investigate the underlying mechanisms of the gene’s effects. In terms of practical implications, the identified SNPs in the MTNR1A gene may be useful for selecting and breeding sheep with desirable reproductive traits. For example, ewes with certain genotypes may be preferred for earlier age at first lambing or higher fertility, while rams with specific alleles may have superior reproductive abilities or mating behavior. Additionally, the use of genetic markers and genotyping techniques can aid in the identification and preservation of rare or valuable sheep breeds.

## Conclusions

In summary, this review underscores the crucial role of the MTNR1A gene in sheep reproduction, presenting opportunities for genetic improvement in various breeds. Extensive studies on MTNR1A SNPs reveal associations with litter size, emphasizing its significance in male reproduction and fecundity. Limited but noteworthy correlations with reproductive attributes emphasize the need for ongoing research, particularly in unexplored gene regions like exon 1. Understanding these mechanisms is vital for optimizing breeding strategies and advancing sheep reproductive outcomes.

## Data Availability

No data are associated with this article.
