# Supplementary material for: Association of melatonin receptor 1 A with litter size in sheep: A review
Source: F1000Res. 2024 Jan 22;12:900. Originally published 2023 Jul 28. [Version 2] doi: 10.12688/f1000research.134890.2 (PMC10844802; doi:10.12688/f1000research.134890.2)
Supplement: Peer review history

# Reviewer report: Reviewer response for version 2

**DOI:** 10.5256/f1000research.161036.r239571

**Published:** 2024-02-05

**Authors:** Ugbo Emmanuel Nnabuike

**Affiliations:** 1 Department of Applied Microbiology, Faculty of Science, Ebonyi State University, Abakaliki, Ebonyi, Nigeria

The authors have made the corrections pointed out for this manuscript. I hereby approve.

Is the review written in accessible language?

Yes

Are all factual statements correct and adequately supported by citations?

Yes

Are the conclusions drawn appropriate in the context of the current research literature?

Yes

Is the topic of the review discussed comprehensively in the context of the current literature?

Yes

Reviewer Expertise:

Medical Microbiology/Epidemiology

I confirm that I have read this submission and believe that I have an appropriate level of expertise to confirm that it is of an acceptable scientific standard.

# Reviewer report: Reviewer response for version 2

**DOI:** 10.5256/f1000research.161036.r239572

**Published:** 2024-01-31

**Authors:** Wenjing You

**Affiliations:** 1 Zhejiang University, Hangzhou, China

All comments have been addressed. I have no more questions.

Is the review written in accessible language?

Yes

Are all factual statements correct and adequately supported by citations?

Partly

Are the conclusions drawn appropriate in the context of the current research literature?

Yes

Is the topic of the review discussed comprehensively in the context of the current literature?

Yes

Reviewer Expertise:

Animal nutrition; metabolic disease

I confirm that I have read this submission and believe that I have an appropriate level of expertise to confirm that it is of an acceptable scientific standard.

# Reviewer report: Reviewer response for version 1

**DOI:** 10.5256/f1000research.147981.r220370

**Published:** 2023-11-21

**Authors:** Wenjing You

**Affiliations:** 1 Zhejiang University, Hangzhou, China

Abuzahra et al. aimed to review the latest literature on the melatonin receptor 1A (MTNR1A) gene as a potential candidate gene associated with reproductive traits, particularly the litter size trait in sheep, by searching various databases for available literature. While the subject addressed is important and interesting, further details need to be clarified.

 The focus should be on melatonin and its receptor MTNR1A, whose roles, sources, structure, and characteristics are little described. Additional information regarding these aspects is needed.

 The authors should also consider describing the mechanisms involved in the production and secretion control of melatonin by the pineal gland, as well as provide an in-depth review of the mechanism by which melatonin regulates the release of follicle-stimulating hormone and luteinizing hormone through the hypothalamic-pituitary-gonadal axis, thereby regulating seasonal reproductive activity in mammals.

 The MTNR1A gene has different effects on reproductive traits in different sheep breeds and sexes, therefore, the section reviewing the genetic relevance of the MTNR1A gene in sheep breeds should be more logical and organized.

 Please provide the full name of the abbreviation, such as SNP, BW1, MM.

Is the review written in accessible language?

Yes

Are all factual statements correct and adequately supported by citations?

Partly

Are the conclusions drawn appropriate in the context of the current research literature?

Yes

Is the topic of the review discussed comprehensively in the context of the current literature?

Yes

Reviewer Expertise:

Animal nutrition; metabolic disease

I confirm that I have read this submission and believe that I have an appropriate level of expertise to confirm that it is of an acceptable scientific standard, however I have significant reservations, as outlined above.

# Reviewer report: Reviewer response for version 1

**DOI:** 10.5256/f1000research.147981.r220362

**Published:** 2023-11-20

**Authors:** Ugbo Emmanuel Nnabuike

**Affiliations:** 1 Department of Applied Microbiology, Faculty of Science, Ebonyi State University, Abakaliki, Ebonyi, Nigeria

The article addressed the purpose of the topic in the area of the function of MTNR 1A in the reproduction performance of animals. I hereby approve for publication. 

 I also suggest that the below comments should be addressed.

 Topic should be corrected to be (Association of melatonin receptor 1 A with litter size in sheep: A review).

 The sub-heading (MTNR 1 A gene and Reproductive Performance of Rams)

 Paragraph 2 (Recent studies, should be corrected to Studies). Since the Reference (35; is 2016 year) so it is not recent.

 I suggest you add all the conclusion together, not concluding in some of the sub-heading.

 Pull all the conclusion together to be in the conclusion part.

Is the review written in accessible language?

Yes

Are all factual statements correct and adequately supported by citations?

Yes

Are the conclusions drawn appropriate in the context of the current research literature?

Yes

Is the topic of the review discussed comprehensively in the context of the current literature?

Yes

Reviewer Expertise:

Medical Microbiology/Epidemiology

I confirm that I have read this submission and believe that I have an appropriate level of expertise to confirm that it is of an acceptable scientific standard, however I have significant reservations, as outlined above.
